# Characterization and expression analysis of *SnRK2*, *PYL*, and *ABF/ AREB/ ABI5* gene families in sweet potato

**DOI:** 10.1371/journal.pone.0288481

**Published:** 2023-11-03

**Authors:** Sarah R. Mathura, Fedora Sutton, Valerie Bowrin

**Affiliations:** 1 Biochemistry Research Laboratory (Rm 216), Department of Life Sciences, The University of the West Indies, St. Augustine, Trinidad and Tobago; 2 ScienceVisions Inc., Brookings, South Dakota, United States of America; University of Delhi, INDIA

## Abstract

Abscisic acid (ABA) signaling in plants is essential to several aspects of plant development, such as tolerance to environmental stresses and growth. ABA signaling is also important for storage organ formation in crops, such as sweet potato. However, the repertoire of *I*. *batatas* ABA signaling gene families has not yet been fully characterized, so that it is unclear which members of these families are necessary for tuberization. Therefore, genome-wide identification of the sweet potato *ABF/ AREB/ ABI5*, *SnRK2*, and *PYL* gene families was performed, along with phylogenetic, motif, *cis*-regulatory element (CRE), and expression analyses. Nine *ABF*, eight *SnRK2*, and eleven *PYL* gene family members were identified, and there was high sequence conservation among these proteins that were revealed by phylogenetic and motif analyses. The promoter sequences of these genes had multiple CREs that were involved in hormone responses and stress responses. *In silico* and qRT-PCR expression analyses revealed that these genes were expressed in various tissues and that *IbABF3*, *IbABF4*, *IbDPBF3*, *IbDPBF4*, *IbPYL4*, *IbSnRK2*.*1*, and *IbSnRK2*.*2* were significantly expressed during storage root development. These results are an important reference that can be used for functional validation studies to better understand how ABA signaling elicits storage root formation at the molecular level.

## Introduction

Abscisic acid (ABA) is a major plant phytohormone that is involved in diverse processes which include growth, development, and adaptation [1]. For instance, ABA is involved in seed development and germination, the adaptive response to environmental stressors (such as temperature extremes, salinity and drought, UVB radiation, and pathogens), flowering, vegetative growth, and seed dormancy [2, 3].

In order for ABA to elicit its effects, various ABA-dependent signalling pathways are activated. Major components of these pathways include: the PYRABACTIN RESISTANCE (PYR/PYL/RCAR) ABA receptors, PROTEIN PHOSPHATASE 2C (PP2C), Sucrose Nonfermenting1-Related Protein Kinase 2 (SnRK2), NADPH oxidases, ion channels and downstream transcription factors (TFs) [3]. One model of ABA-dependent signal transduction suggests that ABA is required to bind to the PYR/PYL/RCAR receptor so that PP2C can bind to the PYR/PYLs and thus inactivate PP2C [3]. This inactivation causes SNF1-type kinases to become active and target TFs to regulate gene expression and ABA-dependent ion channels [3]. TFs bind to *cis*-elements in the promoter regions of ABA-responsive genes [3]. Some of these *cis*-elements include ABA-responsive elements (ABREs), the G-box, CE3, motif III, and hex3 [3].

The (ABA)-responsive element binding proteins/ABRE-binding factors (AREB/ABFs) are a subfamily of transcription factors (TFs) that are involved in the ABA-dependent signalling pathways. AREB/ABFs are the most important TFs involved in the ABA-dependent ABRE signalling pathways [4]. The AREB/ ABF/ ABI5 family of TFs are a subfamily of the bZIP superfamily of proteins. There are two motifs in the bZIP domain: a basic domain that acts as a DNA binding site, and a leucine zipper involved in TF dimerization [4]. These TFs have three conserved regions at the N-terminal, designated as C1, C2, and C3 [4].

The AREB/ ABF/ ABI5 family has been isolated from various plant species so far, including *A*. *thaliana* [2], cotton [5], and potato [3]. In Arabidopsis, 9 members were identified; five ABI5/AtDPBF family genes (ABI5, EEL, DPBF2/AtbZIP67, DPBF4, and AREB3) [6, 7] and four AREB/ABF family genes (AREB1/ABF2, AREB2/ABF4, ABF1, and ABF3) [2]. Similarly, the PYL and SnRK2 families have also been investigated in various crops [8–11]. However, all the members of this pathway in sweet potato (*Ipomoea batatas* (L.) Lam.) are yet to be investigated.

Sweet potato is an economically important crop that is cultivated for its edible root tubers. The mechanism of tuberization in this crop is not fully understood. An enhanced understanding of this process is important to breed better-yielding varieties. ABA has been previously reported to be involved in the development of sweet potato tubers [12]. There is a peak in ABA concentration in the sweet potato root tuber at tuber initiation [13]. Additionally, ABA is involved in various processes during sweet potato tuber development, such as promoting starch deposition [14] and regulating tuber thickening by activating cell divisions in meristems [15]. This raises the question as to whether there are specific ABA signalling genes that are crucial for sweet potato tuber initiation.

In sweet potato, homologs of the AREB/ABF family were found to be up-regulated during drought conditions [16] but their roles in tuberization have not been thoroughly investigated. Dutt et al. [17] noted that *StABF2* and *StABF4* are potential genes that can be overexpressed by genetic engineering to improve potato tuber yield. Two *ABF* genes (*AtABF2* and *AtABF4*) were shown to positively regulate tuber induction in transgenic potato plants, but only *StABF4* was found to improve tuber yield without stunting growth in other plant parts [18]. Additionally, *StABF1* expression was found to increase under tuberizing conditions in potato [19]. In potato (*Solanum tuberosum*), *AtABF4*-expressing plants had increased tuber quality and quantity [20]. Furthermore, StABI5-like 1 (StABL1) promotes early potato tuber maturity via interactions with FLOWERING LOCUS T (FT) homologs and regulation of *GA 2-oxidase 1* genes [21]. It is therefore important to investigate whether the sweet potato homologs of these genes have similar functions during storage root formation.

This paper is the first report of the characterization of the *PYL* and *SnRK2* gene families in sweet potato, and the expression analyses of these families and the *AREB/ ABF/ ABI5* subfamily during storage root formation. The results of this study serve as a basis for the further study of ABA signalling in sweet potato and can be used for further study to improve breeding programs. The most promising gene candidates from this study can be functionally validated in the future to better understand the roles of ABA signalling during storage root initiation and how this impacts yield.

## Materials and methods

### Plant material

Whole sweet potato root tubers (cultivar O49) were obtained from the germplasm collection at The University of the West Indies Field Station (St. Augustine, Trinidad and Tobago). This cultivar is a popular one that is grown in Trinidad and Tobago [22] and the characteristics of this cultivar during storage root initiation and bulking have been previously described [23]. These root tubers were planted singly in 25 cm diameter pots in sandy soil and left for seven weeks. The plants were watered during the day with 500 mL water as required. The plants were fertilized with NPK (12:24:12) fertilizer as directed by the manufacturer. Samples of the storage roots, pencil roots, fibrous roots, leaves, and green stems were harvested at 49 days after transplantation (DAT). Storage root samples at 35 DAT were also collected. Three biological replicates were collected for each tissue. All the samples were used immediately after harvesting.

### Identification of ABA signalling genes in *I*. *batatas* genome

The bioinformatics analyses were conducted on the Galaxy server (https://usegalaxy.eu/) unless otherwise specified [24]. All genes were named according to their homology to the *A*. *thaliana* genes, followed by Chromosome location.

#### *AREB/ABF/ABI5* gene family

The nine AREB/ ABF/ ABI5 *A*. *thaliana* protein sequences were obtained from the UniProtKB database [25] (https://www.uniprot.org/) and were used as query sequences against the sweet potato proteome [26] (www.sweetpotao.com) in a BLASTP search [27] with a threshold e-value of 1e^-10^. The putative protein sequences were examined in the NCBI CDD [28] for the presence of a complete bZIP domain (cd14707: bZIP_plant_BZIP46). The protein sequences were also examined in InterPro [29] (https://www.ebi.ac.uk/interpro/search/sequence-search) for the bZIP domain (IPR043452) and the presence of a DNA binding site and the dimer interface that is characteristic of these proteins (cd14707). The 11 resulting sequences were searched against the nr BLASTP database and any hits that corresponded to G-box binding factor (GBF) proteins were excluded.

#### *PYL* and *SnRK2* gene families

The 14 *A*. *thaliana* PYL and 10 SnRK2 protein sequences were used as queries in a BLASTP search against the sweet potato proteome with e-value of 0.001 and the top 20 hits for each sequence were displayed. The hits were checked for complete domains in the CDD and any hits with the wrong domain were discarded. The SnRK2 hits were also checked in PfamScan (https://www.ebi.ac.uk/Tools/pfa/pfamscan/) for the presence of the protein kinase domain (PF00069). PfamScan was used to check that the PYL proteins had the typical PYL domain (PF10604). Any hits with incomplete domains were used as TBLASTN queries in the *I*. *batatas* NCBI Transcriptome Shotgun Assembly (TSA) database to find complete open reading frames for the protein.

### Protein characterization, phylogenetic analysis, and motif identification

The molecular weight (MW) and pI of the predicted proteins were calculated using the Galaxy Calculate Protein Properties tool [30]. The subcellular location of the proteins was predicted using Plant-mPloc [31] (http://www.csbio.sjtu.edu.cn/bioinf/plant-multi/). The gene intron-exon structure was analysed using GSDS v.2 [32] (http://gsds.cbi.pku.edu.cn/). The conserved motifs in the protein sequences were analysed using MEME v. 5.5.1 [33] (http://meme-suite.org/tools/meme) using default parameters except for the following: any number of repetition were allowed; a maximum of 10 motifs were selected and motif width was set from 6–50. The motifs in the sequences were annotated using InterProScan (https://www.ebi.ac.uk/interpro/search/sequence-search).

Neighbour-joining (NJ) phylogenetic trees were constructed for each gene family using the amino acid sequences of *A*. *thaliana* (downloaded from UniprotKB), *S*. *tuberosum* (downloaded from http://solanaceae.plantbiology.msu.edu/), *Oryza sativa* [10, 34], *I*. *batatas*, *I*. *triloba*, and *I*. *trifida* (downloaded from http://sweetpotato.plantbiology.msu.edu/) using MEGA 11 [35] with 1000 bootstrap replicates.

### Analysis of *cis*-acting regulatory elements in promoter sequences

The 2 kb region upstream of the gene sequences were extracted using bedtools [36] and were analysed in PlantCARE [37] for the presence of *cis*-acting regulatory elements (CREs) (http://bioinformatics.psb.ugent.be/webtools/plantcare/html/). Only hits from the sense strand were accepted.

### *In silico* RNA-Seq expression analysis of ABA signalling genes

Raw RNA-seq reads were downloaded from the NCBI Sequence Read Archive (SRA) (https://www.ncbi.nlm.nih.gov/Traces/study/) and the National Genomics Data Center (NGDC) (https://ngdc.cncb.ac.cn/). The raw data (PRJCA000640) from Ding et al. [38] was downloaded to investigate the expression of the ABA signalling genes in various tissues. The raw data (PRJNA491292) from Wu et al. [39] was used to compare the expression of the ABA signalling genes in storage roots vs. fibrous roots at 30, 40, and 50 days after transplantation (DAT). The time-course datasets (PRJNA647694) of sweet potato roots at different stages of development (fibrous roots [stages S4 and S8], pencil roots [stages S10 and S12], and initiating tuberous roots [stages S14 and S16]) were also used [40]. To investigate the effects of ABA treatment, salicylic acid (SA) treatment, and methyl jasmonate (MeJA) treatment relative to a control, the PRJNA511028 dataset was downloaded from the NCBI SRA. The raw reads were aligned to the genome with STAR [41] and the read counts were obtained from featureCounts [42]. Differential expression was analysed with DESeq2 [43]. The FPKM values were plotted on a heatmap using TBTools [44].

### RNA isolation and qRT-PCR expression analysis

Total RNA was isolated from the plant tissue using the second protocol described by Gromadka et al. [45] with the modification that standard acidified phenol-chloroform RNA extraction was used. The RNA pellets were dissolved in 100% formamide and stored at -20°C. RNA quantity and quality were checked on a Nanodrop 2000 spectrophotometer (Thermo Scientific) and via agarose gel electrophoresis. First strand cDNA synthesis was performed with the SuperScript IV Reverse Transcriptase Kit (Invitrogen, Carlsbad, CA, USA) according to the manufacturer’s directions. Primers were designed using IDT PrimerQuest (S1 Table) (https://www.idtdna.com/pages/tools/primerquest) and synthesized by Macrogen Inc. (Seoul, Korea). The genes that were investigated were chosen based on their significant differential expression in the *in silico* datasets.

The cDNA was diluted twofold and 1 μL of cDNA from three pooled biological replicates was used in each qRT-PCR reaction. The 50 μL qRT-PCR reaction mixture had final concentrations of 1X Power SYBR^®^ Green Master Mix (Invitrogen, Carlsbad, CA, USA), 200 nM forward primer, and 200 nM reverse primer. Each reaction was run in triplicate on a qTower3 thermal cycler (Analytic Jena). The expression levels were normalized to that of the *COX* housekeeping gene [46] and analysed using the 2^-ΔCT^ method. Statistical analyses (One-way ANOVA followed by Duncan’s multiple range post-hoc test (*p*-value ≤ 0.05)) were conducted with IBM SPSS Statistics version 29.

### Prediction of ABA signalling PPI network, Gene Ontology analysis, and KEGG enrichment

The sweet potato proteome in the study was annotated for protein-protein interactions (PPIs) in the STRING-db [47] (https://string-db.org/). The predicted PPI network for the ABA signalling genes were extracted from this network. The ABA signalling proteins were submitted to ShinyGO v. 0.77 (http://bioinformatics.sdstate.edu/go/) and KOBAS-i (http://kobas.cbi.pku.edu.cn/) for Gene Ontology (GO) and KEGG analyses, respectively. The *Ipomoea triloba* and *Ipomoea nil* database annotations were used for each analysis, respectively.

## Results

### Identification of *I*. *batatas* ABA signalling genes

The *I*. *batatas* ABA signalling genes that were identified and characterized in this study are shown in Table 1. The genes were named based on their sequence similarity with the *A*. *thaliana* sequences and by ascending order along the chromosome. Nine *IbABF/ AREB/ ABI5* sequences were identified on six chromosomes with 2–9 exons, pIs ranging from 4.58–9.49, protein MWs ranging from 21.41–50.34 kDa, and they were all predicted to be found in the nucleus.

**Table 1:** Summary of the properties of the sweet potato *AREB/ ABF/ ABI5*, *PYL*, and *SnRK2* gene families.

Name	Gene ID	Chromosome Location	Chromosome Position	Strand	CDS (bp)	Polypeptide Length (a.a.)	MW (kDa)	pI	Subcellular Location	No. of exons
**IbABF1**	g43242	LG11	12787907–12791895	+	1146	381	40.58	9.34	nucleus	8
**IbABF2**	g60407	LG15	3349447–3357226	-	1251	416	44.04	9.46	nucleus	5
**IbABF3**	g38687	LG10	3502437–3506998	+	1410	469	50.34	9.44	nucleus	4
**IbABF4**	g59676	LG14	29831580–29834715	-	1266	421	46.01	9.49	nucleus	4
**IbABF5**	g34305	LG9	1872460–1878103	-	1317	438	47.58	9.11	nucleus	9
**IbDPBF1/ABI5**	g58174	LG14	20423191–20425753	+	1293	430	46.71	8.90	nucleus	5
**IbDPBF2**	g11068	LG3	12140186–12141749	-	591	196	21.41	4.58	nucleus	2
**IbDPBF3**	g58697	LG14	24047044–24052112	-	1302	433	47.56	4.84	nucleus	5
**IbDPBF4**	g24670	LG6	27981131–27985055	+	879	292	31.69	6.98	nucleus	4
**IbPYL1**	g14758	LG4	14690079–14695208	+	654	217	24.65	9.88	cytoplasm, nucleus	1
**IbPYL2**	g20643	LG5	29283472–29286173	-	558	185	20.58	5.80	chloroplast	3
**IbPYL3**	g39321	LG10	8319399–8321665	+	735	244	28.06	6.85	nucleus	4
**IbPYL4**	g20407	LG5	27855575–27856484	-	669	222	24.21	6.72	cytoplasm	1
**IbPYL5**	g24506	LG6	26847908–26849017	+	675	224	24.25	5.50	cytoplasm	1
**IbPYL6**	g25480	LG7	1233155–1234397	+	672	223	24.24	6.62	cytoplasm	2
**IbPYL7**	g26930	LG7	12399720–12402106	-	558	185	20.83	6.16	chloroplast	3
**IbPYL8**	g928	LG1	5425878–5427174	+	627	208	23.61	6.17	nucleus	3
**IbPYL9**	g9406	LG2	37501442–37503256	-	585	194	21.67	5.23	chloroplast	4
**IbPYL10**	g19566	LG5	21751387–21753890	+	708	235	26.97	5.36	nucleus	4
**IbPYL11**	g55788	LG14	3155840–3157156	+	696	231	25.79	5.38	cytoplasm, nucleus	1
**IbSnRK2.1**	g59428	LG14	28625327–28628503	+	1059	352	40.27	5.90	nucleus	9
**IbSnRK2.2**	g20838	LG5	30367121–30370193	-	1083	360	41.25	6.29	nucleus	9
**IbSnRK2.3**	g1128	LG1	6759837–6763761	+	1104	367	41.48	4.70	nucleus	8
**IbSnRK2.4**	g34342	LG9	2079341–2082903	-	1095	364	41.99	5.34	nucleus	9
**IbSnRK2.5**	g8093	LG2	28697148–28701164	-	996	331	36.86	5.54	nucleus	9
**IbSnRK2.6**	g34108	LG9	884554–889989	-	1116	371	41.70	4.67	nucleus	9
**IbSnRK2.7**	g29557	LG7	31078162–31080710	-	1077	358	40.08	4.87	nucleus	10
**IbSnRK2.8**	g42081	LG11	4543461–4546632	-	921	306	35.04	5.38	nucleus	8

**Fig 1:**
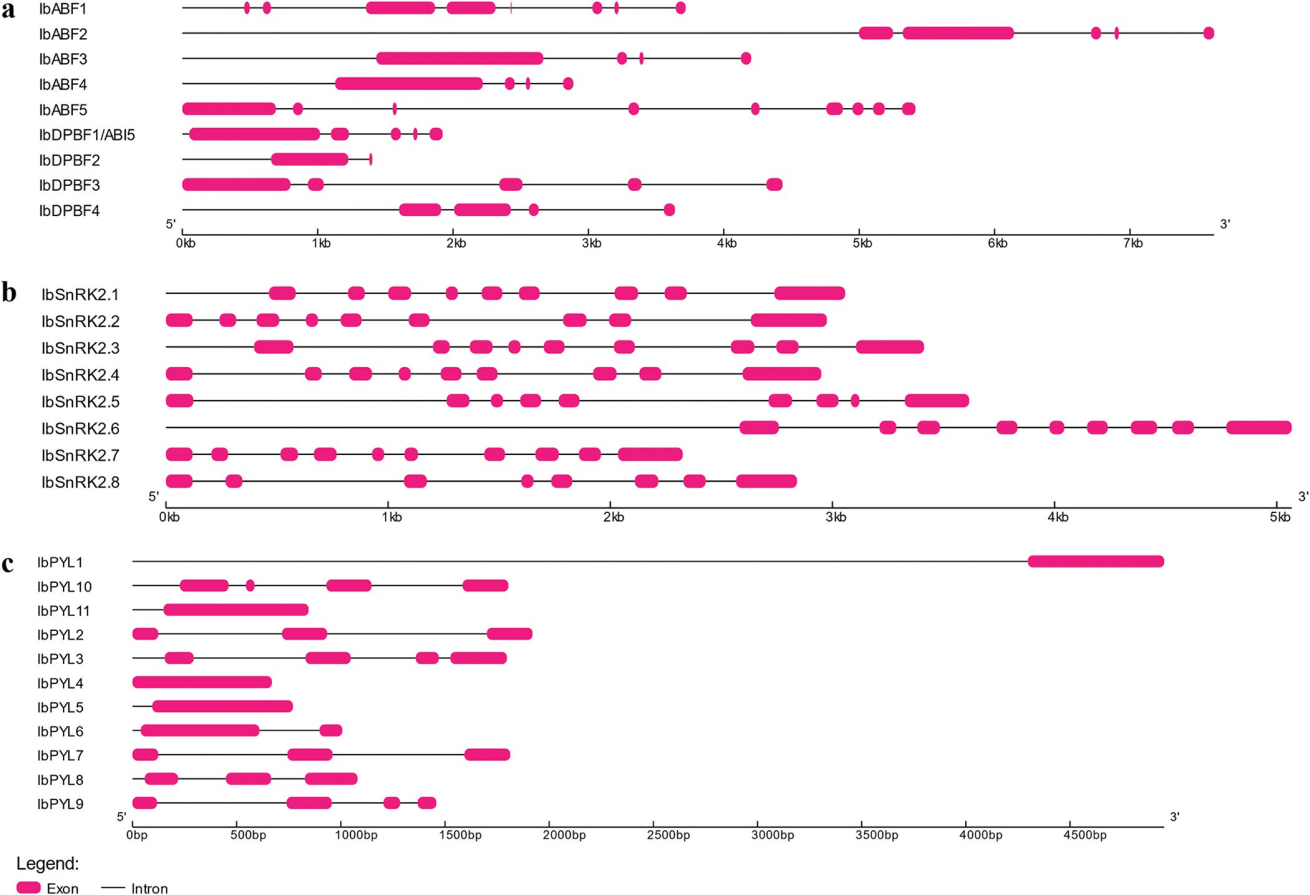
Intron-exon arrangement of sweet potato ABA signalling genes. (**a**) *ABF/ AREB/ ABI5*, (**b**) *SnRK2*, and (**c**) *PYL* gene families.

Twelve *PYL* gene sequences were discovered in BLASTP. Of these 12 sequences, 2 sequences (g9406 and g25480) showed an incomplete domain. TBLASTN of these 2 sequences against the sweet potato transcriptome shotgun assemblies in NCBI was used to yield the corrected versions of these proteins with the full PYL domain (see S1 File for protein sequences used). One sequence (g55782) only yielded hits that were similar to g55788 so this sequence was discarded, giving a final total of 11 *IbPYL* genes (Table 1). These 11 sequences had 1–4 exons (Fig 1), and the proteins encoded by these genes ranged from 185–244 a.a. in length, and were predicted to localize to the nucleus, chloroplast, and cytoplasm.

The eight *IbSnRK2* genes had coding sequences comprised of 8–10 exons, and these sequences encoded proteins that were 306–371 a.a. in length, had a narrow pI range from 4.67–6.29, and were all predicted to localize in the nucleus.

### Motif analysis of *I*. *batatas* ABA signalling proteins

**Fig 2:**
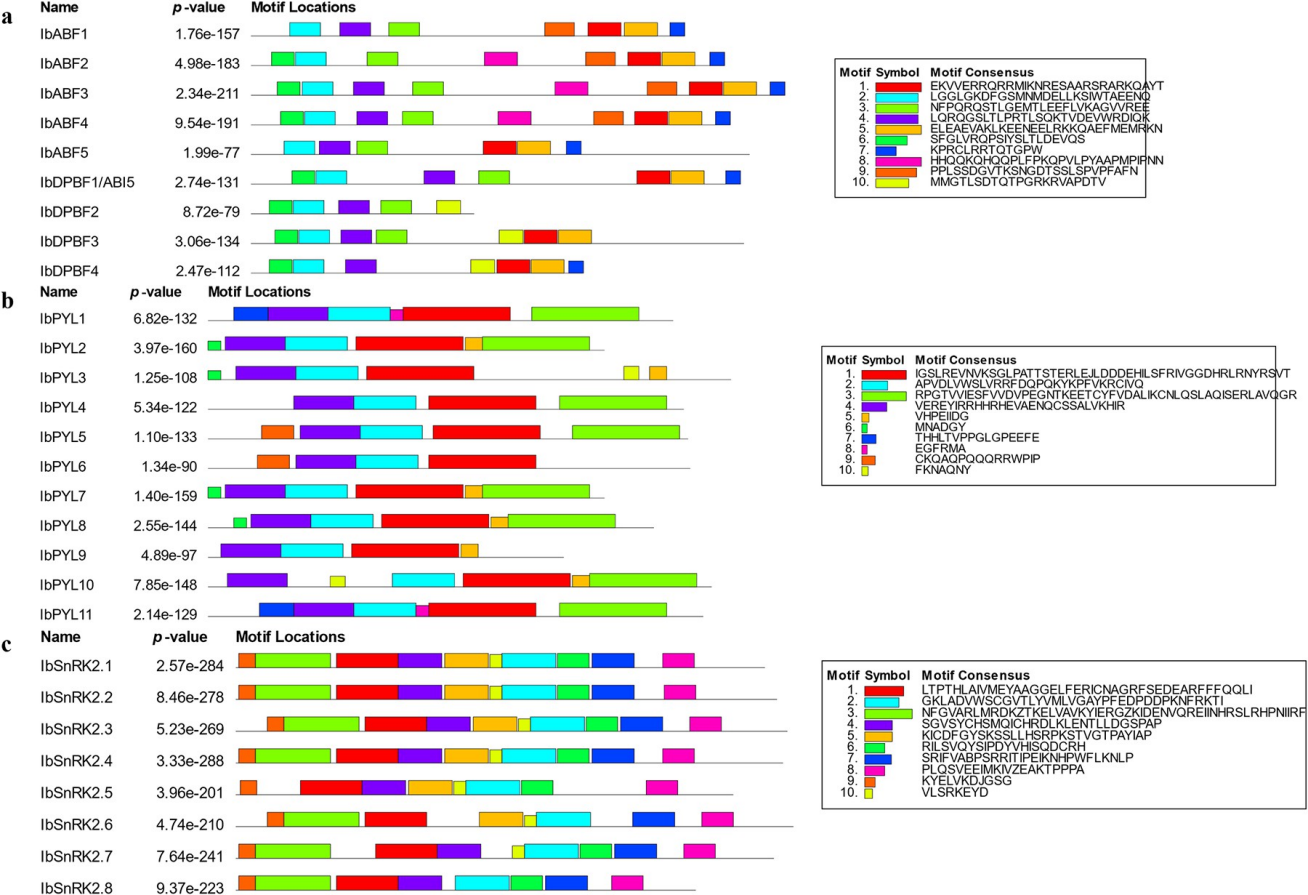
Motifs in the ABA signalling proteins discovered using MEME. The motif arrangements are shown for the (**a**) ABF/ AREB/ ABI5 family, (**b**) PYL family, and (**c**) SnRK2 family. A maximum of 10 motifs were searched with motif length between 10–50 residues. Motif occurrence was set to zero or one occurrence per sequence.

The results of the MEME analysis to find conserved motifs in the *I*. *batatas* ABA signalling proteins are depicted in Fig 2. There were 10 conserved motifs found in the ABF/ AREB/ ABI5 proteins (Fig 2A) and the motif order was conserved among the sequences. Motif 2 was the only motif present in all nine IbABF sequences. All nine sequences had a combination of one or more of Motifs 1, 3, and 4. Motif 1 represents the IPR043452 bZIP domain that is characteristic of these TFs.

Ten conserved motifs were found in the IbPYL proteins and there were similar arrangements of these motifs in the sequences (Fig 2B). Motifs 1, 2, and 4 were present in all the IbPYL proteins and these motifs, along with Motif 3, represent the START-like domain (IPR023393).

There were also ten conserved motifs with similar arrangements in the IbSnRK2 proteins (Fig 2C). Motifs 1, 2, 8, and 9 were present in all 8 IbSnRK2 proteins. Motifs 1–5, and Motif 7 represent the protein kinase domain (IPR011009) that is characteristic of these proteins.

### Phylogenetic analysis of *I*. *batatas* ABA signalling proteins

**Fig 3:**
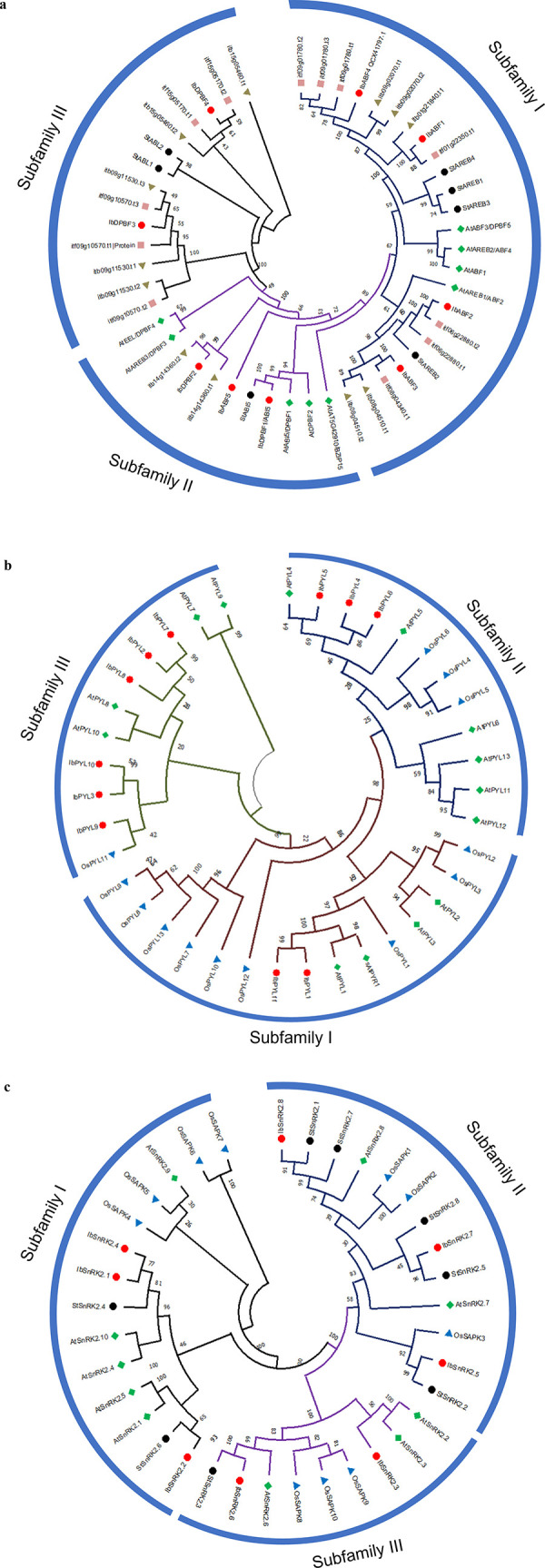
Neighbour-joining phylogenetic trees of ABA signalling gene families. The phylogenetic trees were constructed for the (**a**) ABF/ AREB/ ABI5, (**b**) PYL, and (**c**) SnRK2 protein sequences. The percentage of replicate trees in which the associated taxa clustered together in the bootstrap test (1000 replicates) are shown next to the branches. Ib: *I*. *batatas*; St: *S*. *tuberosum*; itf: *I*. *trifida*; itb: *I*. *triloba*; At: *A*. *thaliana*; Os: *Oryza sativa*.

The IbABF/ AREB/ ABI5 protein sequences clustered into three phylogenetic subfamilies (Fig 3A), with 4, 3, and 2 IbABF/ AREB/ ABI5 sequences in Subfamilies I, II, and III respectively. Subfamily I members had the unique Motif 9 in their sequences.

The IbPYL protein sequences were grouped into three subfamilies in a phylogenetic tree (Fig 3B) with 2, 3, and 6 IbPYLs belonging to Subfamilies I, II, and III respectively. IbPYLs with similar domain organization clustered in the same subfamily. For example, IbPYL1 and IbPYL11 from Subfamily 1 have the unique Motif 8 (Fig 2B), while IbPYL-2, -3, -7, -8, -9, and -10 belonged to Subfamily III and contained the unique Motif 5. Subfamilies I and II have IbPYLs that were encoded by genes with one or two exons only (Fig 1) while Subfamily III IbPYLs were encoded by genes with three or four exons only.

The IbSNRK2 proteins were grouped into three phylogenetic subfamilies I, II, and III (Fig 3C), with 3, 3, and 2 IbSnRK2s respectively. Subfamily I contained IbSnRK2 proteins that were encoded by coding sequences with nine exons, while the coding sequences for members of Subfamilies II and III had 8–10 exons. There were no motifs that were specific to any subfamily (Fig 2C).

### Analysis of CREs in the promoter sequences of ABA signalling genes

The CREs that were identified in the ABA signalling gene sequences are shown in S2 Table. All the ABA signalling genes had multiple light-responsive elements. There were also several CREs present that are involved in hormone responsiveness. The most commonly occurring hormone-responsive CRE was the MeJA-responsive CRE, which was present in 21 of the 28 sequences. Fifteen of these sequences had at least one ABA-responsive CRE (ABRE). Less than half of the sequences had CREs for responsiveness to auxin, GA, SA, and ethylene. There were also several CREs present that are involved in stress responses, development, and protein binding. Some of the common developmental CREs were as-1 (involved in root-specific expression), HD-Zip 1 (involved in palisade mesophyll cell differentiation), and CAT-box (involved in meristem expression). There were numerous CREs involved in stress and defence responses such as ARE (anaerobic induction), DRE core (drought responsiveness), LTR (low temperature responsiveness), MBS (drought inducible), STRE (stress responsiveness), and wound responsiveness (W box, WUN-motif, WRE).

### Analysis of *in silico* ABA signalling gene expression

**Fig 4:**
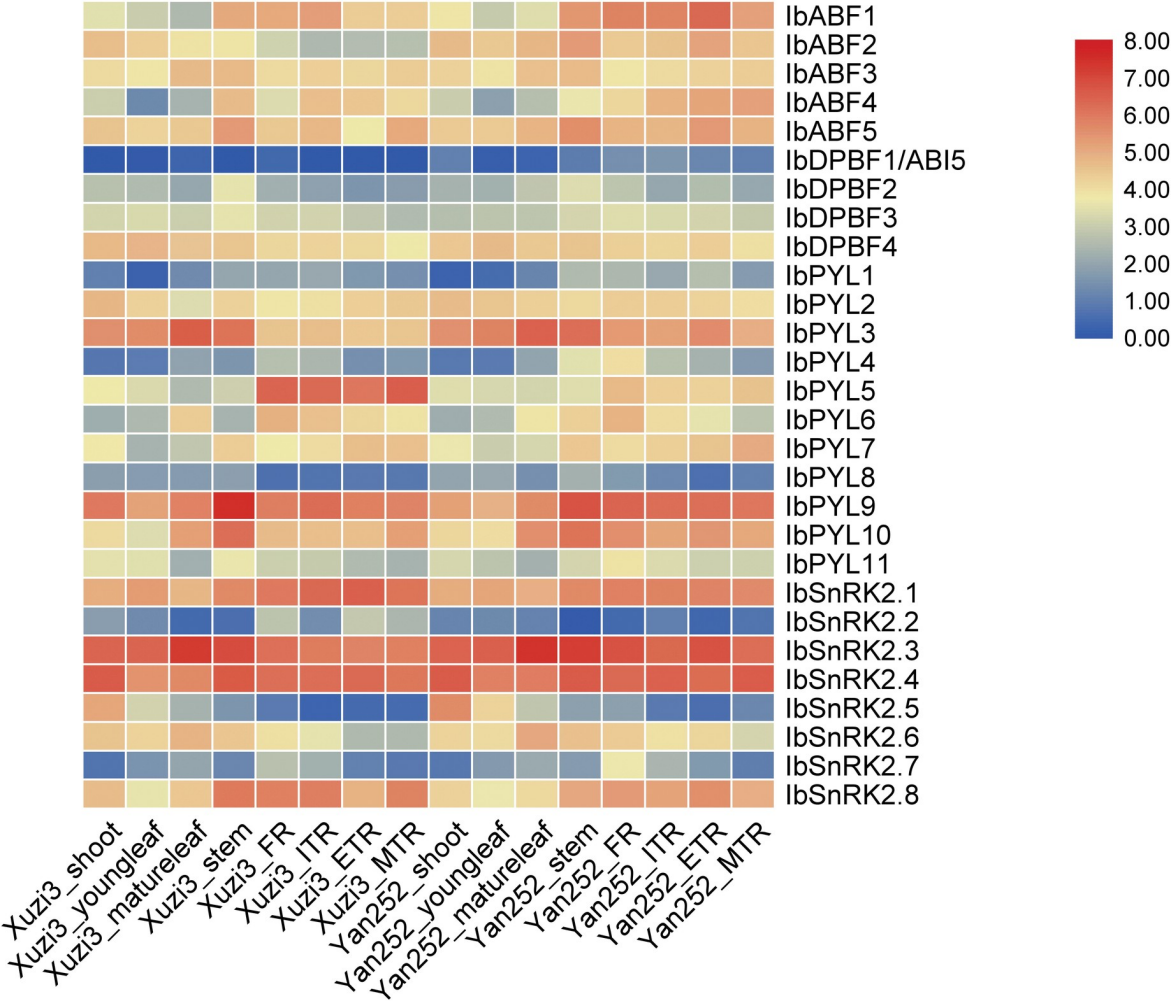
Heatmap illustrating the expression of ABA signalling genes in different sweet potato plant parts. The scale bar represents the log_2_(FPKM +1) expression values. The raw data from Ding et al. [38] was used to obtain the expression levels of the ABA signalling genes in various plant parts (shoot, young leaf, mature leaf, stem, fibrous root [FR], initiating tuberous root [ITR], expanding tuberous root [ETR], and mature tuberous root [MTR]) from two sweet potato cultivars (Xuzi3 and Yan252).

The *in silico* expression of the ABA signalling genes in various sweet potato plant parts in two different cultivars is shown in Fig 4. The expression patterns were similar between the two cultivars. Some genes (*IbPYL9*, *IbSnRK2*.*3*, *IbSnRK2*.*4*) showed high expression regardless of tissue type, while others, such as *IbABI5* and *IbPYL8* showed low expression across different tissue types. *IbPYL3* had highest expression in mature leaves and stems, while *IbSnRK2*.*5* had highest expression in shoots and young leaves. *IbABF1*, *IbABF4*, *IbPYL5*, *IbSnRK2*.*1*, and *IbSnRK2*.*8* have higher expression values in root and/or stem tissue. *IbSnRK2*.*7* had its highest expression in FRs.

**Fig 5:**
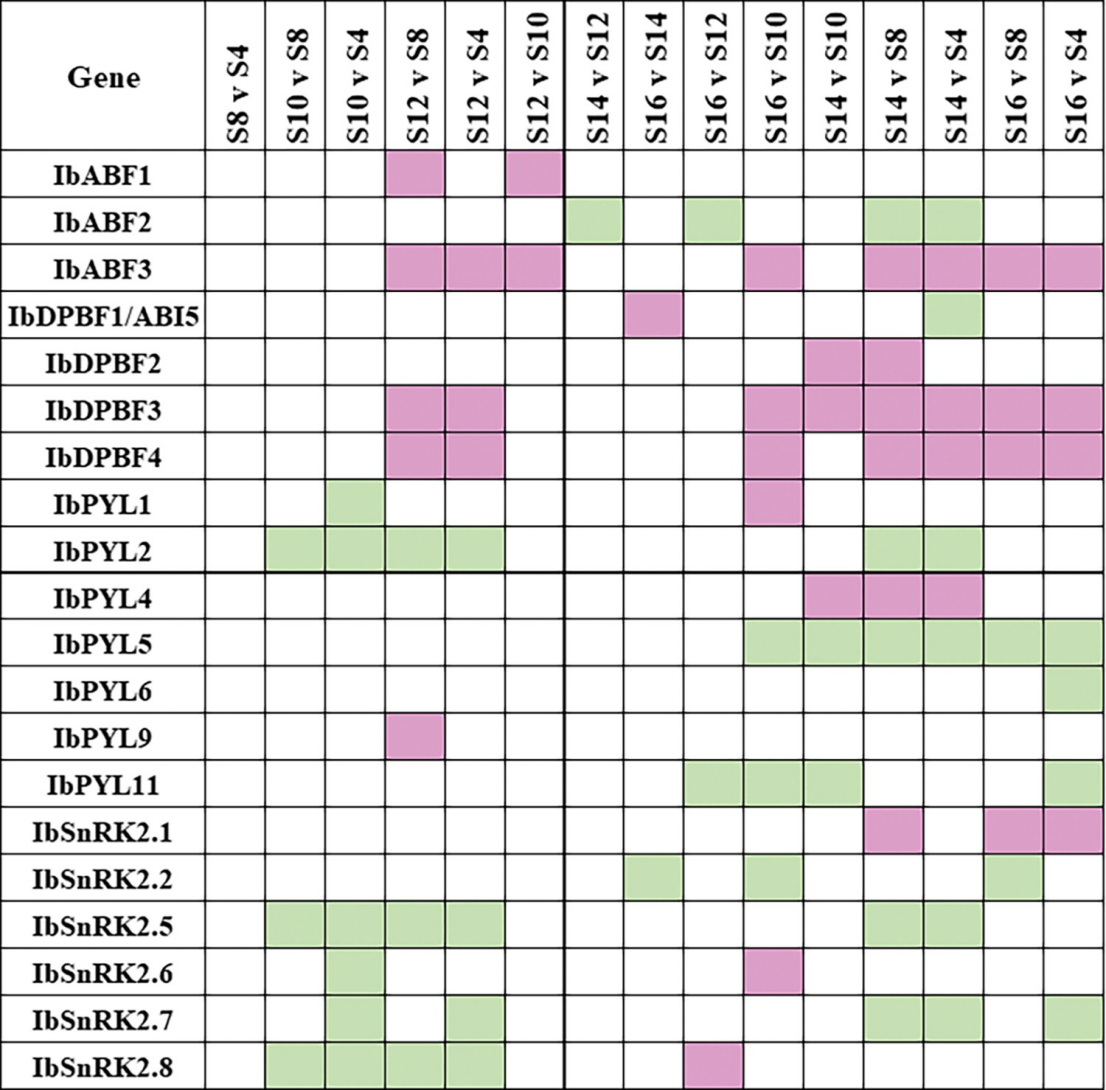
Heatmap illustrating the expression of ABA signalling genes in *I*. *batatas* roots at various stages of development. The heatmap illustrates the DEGs at different time points during sweet potato SR development obtained using RNA-seq data from He et al. [40]. The stages that were compared were fibrous roots (S4, S8), pencil roots (S10, S12), and initiating storage roots (S14, S16). Pink boxes represent up-regulation and green boxes represent down-regulation. There was no significant fold change for the white boxes. Only differentially expressed ABA signalling genes are shown. The criteria for differential expression are |log_2_FC| ≥ 1 and adjusted *p*-value ≤ 0.05.

**Fig 6:**
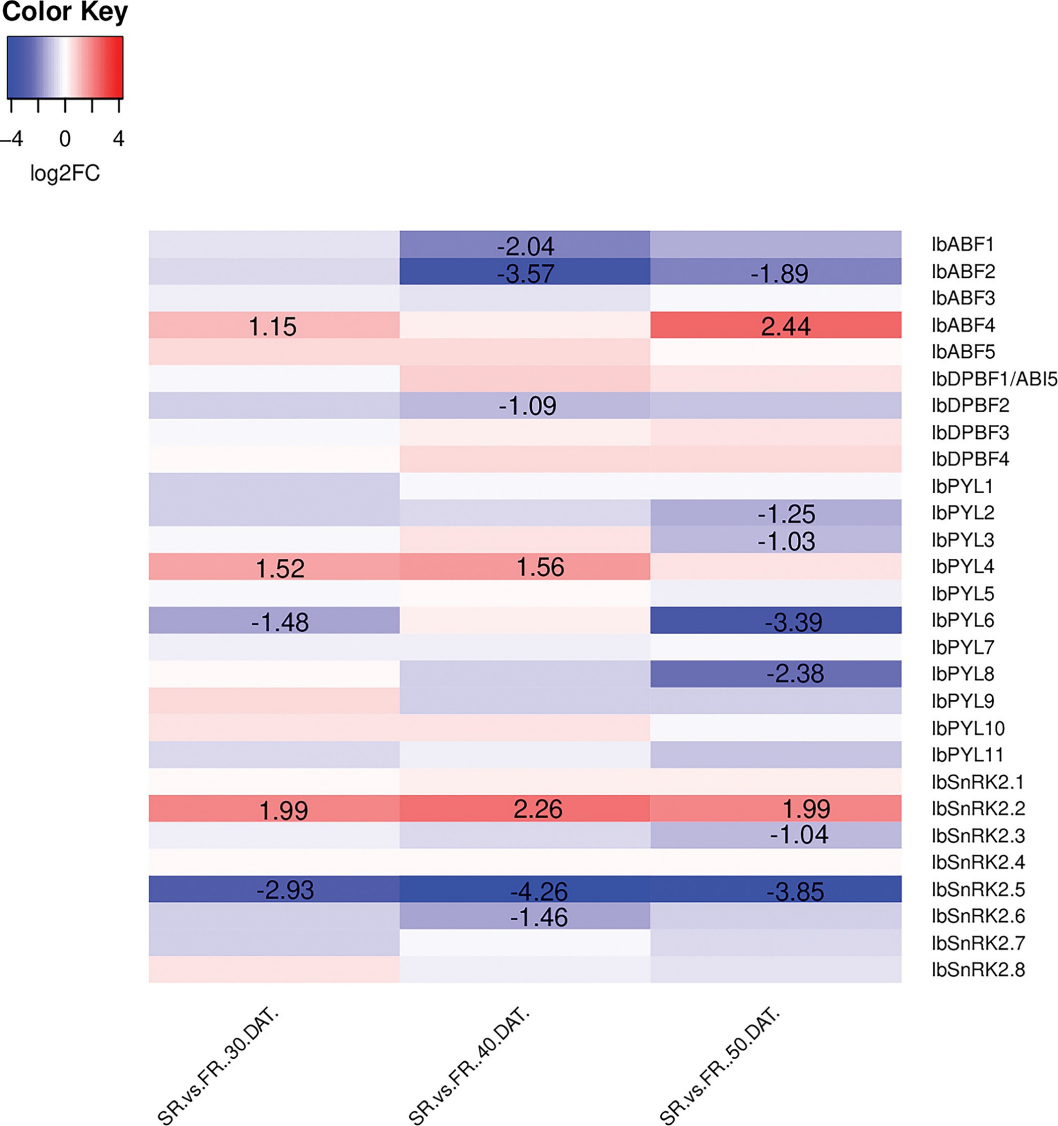
Heatmap showing the expression of ABA signalling genes from fibrous roots and storage roots at different developmental stages. The RNA-seq data from Wu et al. [34] was used to calculate and plot the log_2_FC of ABA signalling genes in SRs vs. FRs at 30, 40, and 50 DAT respectively. Statistically significant fold changes are labelled (*p*-value ≤ 0.05 and log_2_FC ≥ 1). Blue represents down-regulation while red represents up-regulation.

The *in silico* expression of ABA signalling differentially expressed genes (DEGs) involved in SR initiation are illustrated in Fig 5. *IbABF3*, *IbDPBF2*, *IbDPBF3*, *IbDPBF4*, *IbPYL4*, and *IbSnRK2*.*1* are up-regulated as SR initiation progresses, whereas *IbABF2*, *IbPYL2*, *IbPYL5*, *IbPYL11*, *IbSnRK2*.*2*, *IbSnRK2*.*5*, and *IbSnRK2*.*7* are down-regulated. The differentially expressed ABA signalling genes when comparing *in silico* SR vs. FR gene expression as SR initiation progresses are highlighted in Fig 6. There are similarities between the expression of some of the genes in Figs 5 and 6. For example, *IbABF2*, *IbPYL2*, *IbPYL6*, and *IbSnRK2*.*5* are significantly down-regulated and *IbPYL4* is significantly up-regulated in both datasets. There were also some differences, since *IbABF4* and *IbSnRK2*.*2* are up-regulated in Fig 6, but the former is not differentially expressed, and the latter is down-regulated in Fig 5.

**Fig 7:**
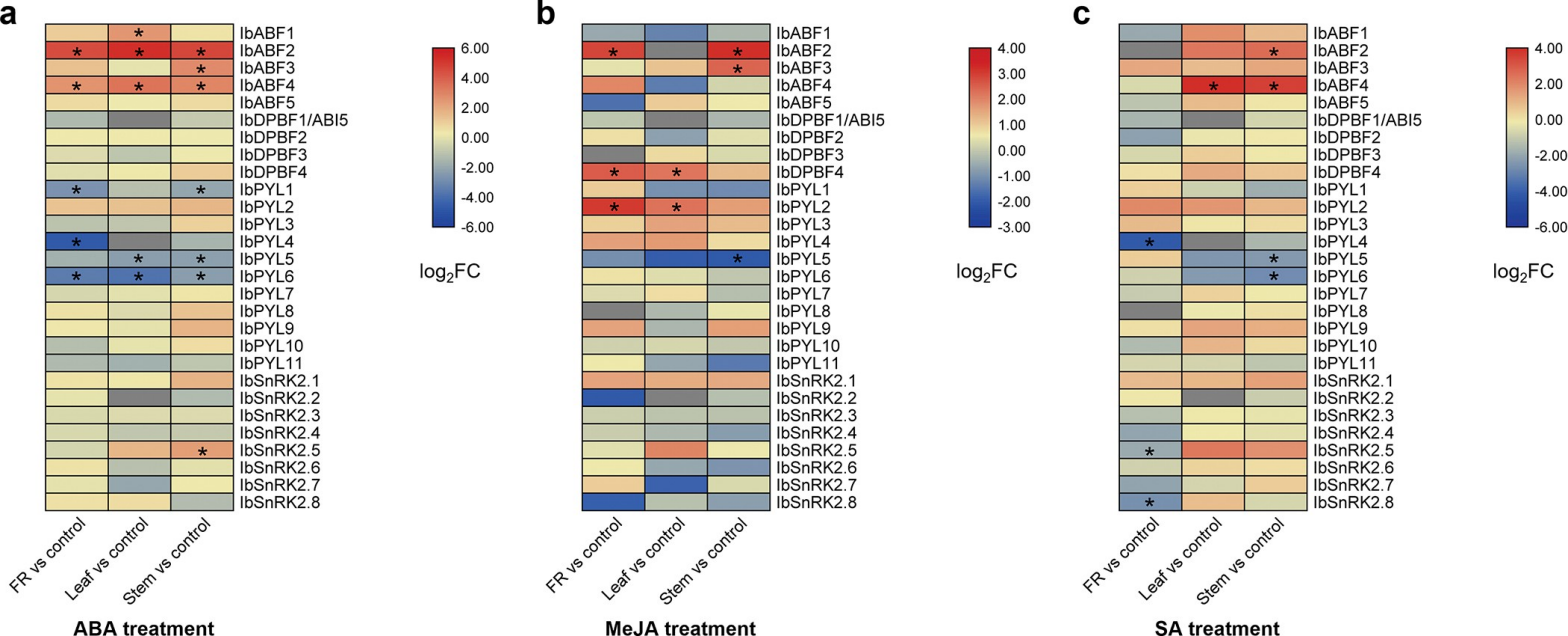
Expression heatmaps showing the fold changes of ABA signalling genes in sweet potato cultivar Xushu18 in response to different hormone treatments. (a) ABA treatment (b) MeJA treatment (c) SA treatment. Differentially expressed genes (DEGs) were identified using the criteria of log_2_FC ≥ 2 and *p*-value < 0.05. DEGs are denoted by an asterisk and genes whose expression was too low to calculate a fold change are shown as grey boxes. Red boxes represent up-regulation while blue boxes represent down-regulation.

The *in silico* expression of the ABA signalling genes in response to the application of ABA, MeJA, and SA in FRs, leaves, and stems of sweet potato cultivar Xushu18 are shown in Fig 7. For each hormone treatment, there was some tissue-dependent variations in the expression patterns. The largest number of DEGs were observed in response to ABA (Fig 7A). *IbABF2* and *IbABF4* were significantly up-regulated in response to ABA treatment in all three plant parts, whereas *IbPYL6* was down-regulated in response to ABA treatment in all three plant parts. *IbABF3* and *IbSnRK2*.*5* were significantly up-regulated in stems in response to ABA while *IbPYL1* and *IbPYL5* were significantly down-regulated in stems after ABA treatment. In leaves, *IbABF1* was significantly up-regulated and *IbPYL5* was significantly down-regulated in response to ABA treatment. In FRs, *IbPYL1* and *IbPYL4* were significantly down-regulated in response to ABA treatment.

Less genes were differentially expressed in response to MeJA (Fig 7B). *IbDPBF4* and *IbPYL2* were significantly up-regulated in response to MeJA treatment in FRs and leaves. In FRs, *IbABF2* was also up-regulated in response to MeJA. In stems, *IbABF2* and *IbABF3* were up-regulated and *IbPYL5* was down-regulated in response to MeJA, just like their responses to ABA in stems.

Compared to the previous two hormone treatments, there were more down-regulated ABA signalling genes in response to SA (Fig 7C). Only *IbABF2* (in the stem) and *IbABF4* (in the leaf and stem) were up-regulated, while *IbPYL5* and *IbPYL6* (in the stem) and *IbPYL4*, *IbSnRK2*.*5*, and *IbSnRK2*.*8* (in the FR) were down-regulated in response to SA. The *ABF/AREB/ABI5* subfamily showed the most up-regulated genes in response to ABA, MeJA, and SA, when compared to that of the *PYL* and *SnRK2* families, which showed down-regulation in some cases.

### qRT-PCR expression analyses of *ABF/AREB/ABI5*, *PYL*, and *SnRK2* genes

**Fig 8:**
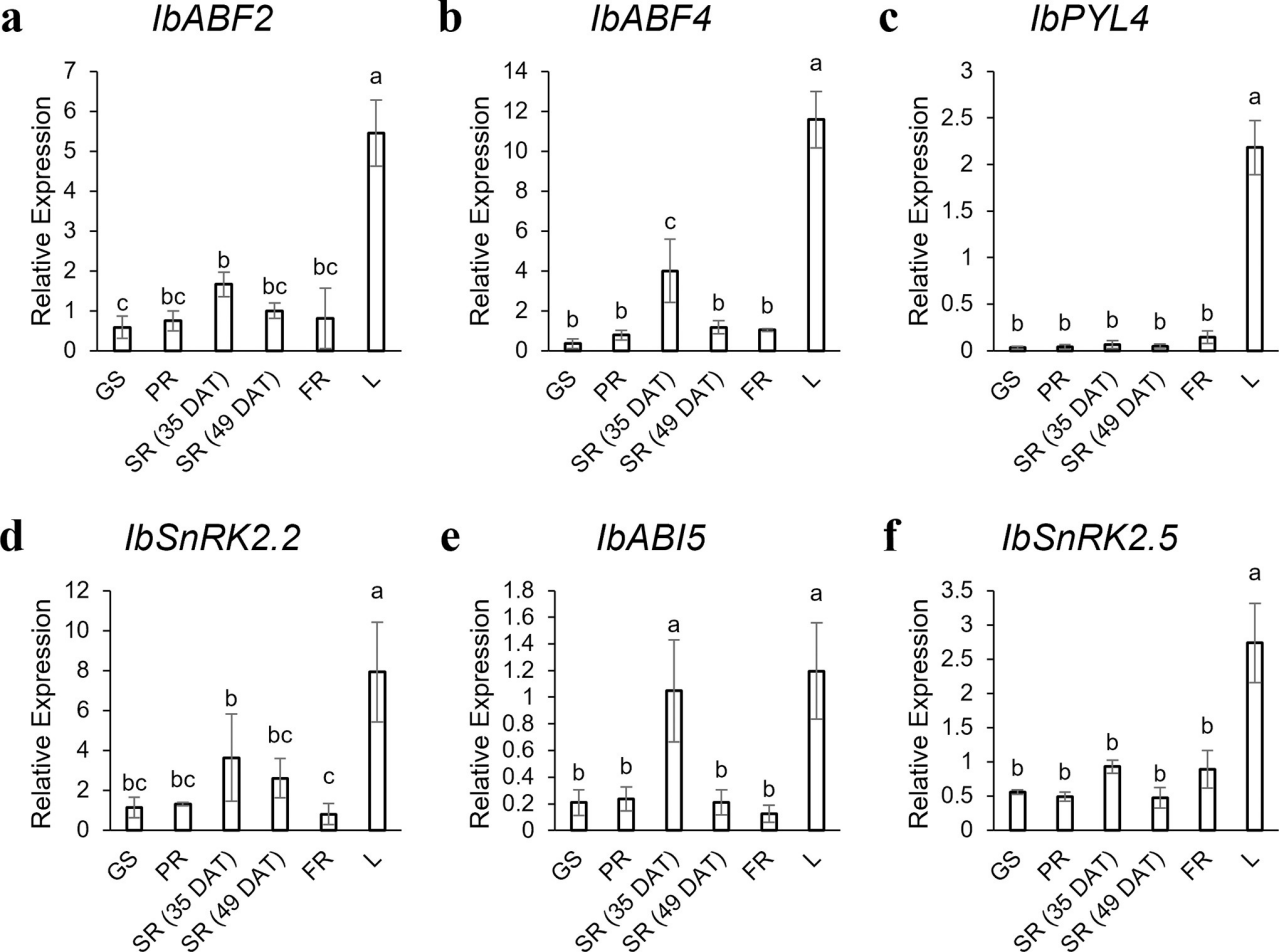
qRT-PCR results of the expression of ABA signalling genes in different *I*. *batatas* tissues. Tissue samples were collected at 49 DAT (unless otherwise stated for the SR sample at 35 DAT). The relative expression was calculated by normalization of the expression values to the *COX* housekeeping gene. Error bars represent the standard deviation of three replicates. Bars not sharing a letter show statistically significant differences (*p*-value ≤ 0.05) using Duncan’s multiple range test. (GS–green stem; PR–pencil root; SR–storage root; FR–fibrous root; L–leaf).

The results of the qRT-PCR expression analyses are illustrated in Fig 8. The expression of the six ABA signalling genes that were investigated were highest in the leaf than in other tissues. The up-regulation of *IbSnRK2*.*2* (Fig 6) and the down-regulation of *IbSnRK2*.*5* in SRs compared to FRs that was observed in the *in silico* data (Figs 5 and 6) were also observed in the qRT-PCR data (Fig 8D and 8F). *IbABI5* had the lowest expression values in the qRT-PCR results for most of the tissues (Fig 8E), and this gene also showed the lowest expression in most of the tissues from the *in silico* dataset (Fig 4). The expression levels of *IbABF2*, and *IbPYL4* were similar in SRs vs. FRs in the qRT-PCR results while there were significant differences in Fig 6. The expression of *IbABF4* at 35 DAT in SRs was significantly higher than that in the other plant parts, excepting the leaf), and this was similar to the higher expression of *IbABF4* in SRs vs. FRs that was seen in Fig 6.

### Predicted PPI network of ABA signalling genes

**Fig 9:**
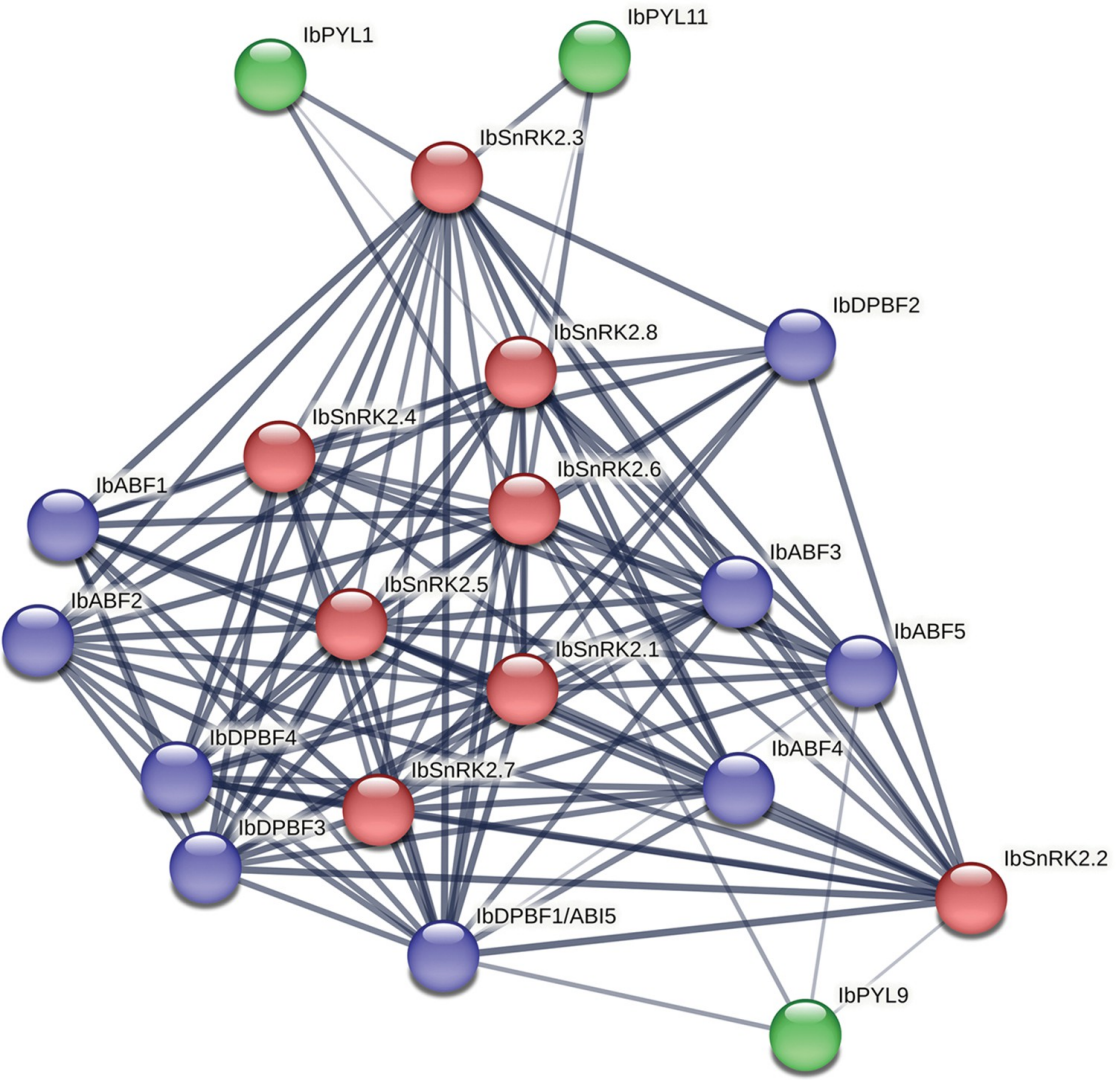
Predicted PPI network for *I*. *batatas* ABA signalling proteins. The nodes are coloured by protein family (blue–ABF/ AREB/ ABI5; green–PYL; red–SnRK2). The edge thickness is directly proportional to the confidence score of the predicted interaction. Only predicted interactions with a minimum medium confidence score of 0.4 are displayed. The disconnected nodes are hidden from the network.

The predicted ABA signalling PPI network that was obtained from STRING is illustrated in Fig 9. A densely connected network was obtained with several of the ABA signalling genes showing high confidence predicted interactions (interaction score > 0.7) with each other, as opposed to individual clusters of genes. While all the IbABF/ AREB/ ABI5 and IbSnRK2 proteins had predicted interactions with each other, only three of the 11 IbPYLs had predicted interactions in the network. These IbPYL proteins (IbPYL-1, -9, and -11) showed high confidence predicted interactions with IbSnRK2.3 and IbSnRK2.6 and medium confidence predicted interactions with IbSnRK2.2, IbSnRK2.8, IbABF5, and IbABI5.

GO analysis revealed that these proteins were annotated to GO terms involved in plant responses to various environmental stimuli (S1 Fig). These GO terms include: ‘regulation of stomatal closure’, ‘response to carbon dioxide’, ‘pollen maturation’, ‘cellular response to light intensity’, ‘response to water deprivation’, ‘response to salt stress’, and ‘response to acid chemical’. The gene list was also enriched in the salt/ drought/ osmotic stress pathway of the ‘MAPK Signaling Pathway–Plant’ KEGG pathway and the ABA signalling pathway in the ‘Plant Hormone Signal Transduction’ KEGG pathway. These annotations lend further support for the predicted roles of these proteins in the plant’s response to environmental stresses.

## Discussion

ABA signalling is vital for several aspects of plant development, including abiotic stress responses, dormancy and germination, regulation of stomatal movements, and vegetative growth [48, 49]. ABA is also involved in the formation and development of stem tubers and root tubers in various crops [50]. Although there has been progress in understanding ABA signalling during tuberization in potato [3], this process is less well-defined in *I*. *batatas*. Therefore, this study characterized the repertoire of the ABF/ AREB/ ABI5, PYL, and SnRK2 gene families to gain a better understanding of how they are expressed during storage organ formation.

### *I*. *batatas* ABA signalling proteins are highly conserved

The intron-exon arrangements of the coding sequences were similar to that of ABA signalling genes from other crops such as potato [3], soybean [11], rice [10], and cotton [8]. The high degree of conservation of these sequences were also reflected in their domain organization and phylogenetic relationships.

The nine IbABF/ AREB/ ABI5 protein sequences clustered together with their *A*. *thaliana* bZIP protein homologs, as also described by Liu et al. [51]. Liu et al. reported 11 IbbZIP Group A members, while we reported 9 members since, as described earlier, we excluded any AtGBF homologs from the results, since GBF proteins are not involved in the ABA signalling pathway. Dröge-Laser et al. [52] identified 13 Group A AtbZIPs, but four of these (AtbZIP-13, -14, -27, and -40) were either uncharacterized, a GBF4 protein, or flowering locus D (and its paralog). Since these four bZIPs are not part of the ABA signalling pathway, they were not used as search queries.

The 11 IbPYL protein sequences were clustered into three subfamilies (Fig 3B) and this phylogenetic arrangement was also observed in other studies [8–10]. The clustering of SnRK2 proteins in grape [53] and sugarcane [54] into three subfamilies is consistent with this study. The IbSnRK2 proteins had a highly conserved N-terminal protein kinase domain, and the C-terminus was more variable, which is consistent with previous reports that the variability of the C-terminus contributes to the functional diversity of these proteins [54].

The SnRK2 proteins contained most or all of the 10 motifs that were identified in this family (Fig 2), indicating that this family was highly conserved. However, none of the members of the PYL family or the ABF/AREB/ABI5 subfamily contained all 10 identified motifs, with few of the members having identical motif arrangements. This dissimilarity in the motifs present amongst these gene family members may be associated with divergence of the sequences that may lead to functional variation.

The similarity of expression of some of the genes from the same phylogenetic subfamily (e.g. *IbDPBF3* and *IbDPBF4*) indicate that there may be some functional redundancy among homologous gene pairs. However, the expression differences between the majority of genes within a subfamily indicate that some of these genes have unique functions, which contribute to the diversity of ABA-mediated responses in the plant.

### ABA signalling during *I*. *batatas* storage organ formation

ABA is an important regulator of storage organ development, but there is a lack of studies that investigate the specific roles of ABA during tuberization [50]. In sweet potato, ABA is involved in cambial activity in the meristem and regulation of starch deposition [12, 14]. Chen et al. [50] summarized the current state of knowledge of the ABA signalling genes during storage organ formation. They found that there was both up- and down-regulation of *ABFs*, *PYLs*, and *SnRK2s* during storage organ formation in lotus (*Nelumbo nucifera*), carrot, radish, and *Panax notoginseng* [50]. There was a lack of corresponding data for crops that form storage roots, such as cassava and sweet potato [50]. Therefore, this study sought to fill this gap in the current understanding of storage root formation.

The up-regulation of several ABA signalling genes during SR formation suggests that these genes are likely to have various roles during the tuberization process. There were some similarities and differences in the expression of these genes in sweet potato and that of their homologs in *S*. *tuberosum*. For example, *StABF1/AREB2* is up-regulated as tuberization progresses [19], but its homolog, *IbABF2*, is down-regulated during SR development (Figs 5 and 6). The significant expression of *IbABF4* during SR formation (Figs 6 and 8) is supported by the finding that *StABF4* overexpression led to increased yield in transgenic potato plants through regulation of ABA-GA crosstalk [18, 20]. Chen et al. [50] discussed how ABA and GA have antagonistic effects that mediate tuberization in several crops. Liu et al. [3] found several genes from the *StABF/ AREB/ ABI5* gene family were up-regulated in potato stolons at the onset of tuberization and similar results are observed for *I*. *batatas*, where *IbABF3*, *IbDPBF2*, *IbDPBF3*, and *IbDPBF4* are up-regulated during SR initiation (Fig 5). *StABL1* also regulates potato tuberization by interacting with the *StSP6A* tuberigen and altering GA metabolism [21]. The up-regulation of the *StABL1* homologs, *IbDPBF3* and *IbDPBF4*, warrants further studies to determine whether these genes promote SR initiation via a mechanism similar to that in potato [21]. There is a paucity of previous studies that determine the functions of PYL and SnRK2 proteins during storage organ formation. Previous research found that the expression levels of Auxin Response Factor 4 (*ARF4*) and *ARF10* genes involved in SR initiation are positively correlated with *MePYL1* and *MeSnRK2*.*6* in cassava (*Manihot esculenta*) and negatively correlated with *MePYL2* [55]. Therefore, the significant up-regulation of *IbPYL4*, *IbSnRK2*.*1*, and *IbSnRK2*.*2* warrant further investigation.

### ABA signalling is involved in sweet potato development

The sweet potato ABA signalling genes have other roles during plant development. The expression of these ABA signalling genes throughout the plant in various plant parts (Fig 4) highlight their importance in various aspects of plant development. An interesting finding was that there was high expression of some of the ABA signalling genes in leaves (Fig 8) although this was not observed for most genes in the *in silico* dataset (Fig 4). This may be due to a cultivar-specific difference or due to the difference in planting conditions.

These ABA signalling genes are important in the sweet potato response to various hormones (Fig 7). Our observation of the stronger responsiveness of *ABF/AREB/ABI5* genes in response to ABA (when compared to that with other hormones) was corroborated by a previous study [51].

Most of the *IbSnRK2* genes did not show differential expression in response to hormone treatments, and this was observed in potato, where *StSnRK2* genes did not show much obvious responses to ABA [56]. The *StSnRK2* genes showed significant changes in response to salt and osmotic stress, so that the *IbSnRK2* genes are likely to be more involved in responses to stress than in responses to hormones. It is interesting to note that *IbSnRK2*.*1* and *IbSnRK2*.*2*, which was up-regulated during SR development (Figs 5 and 6 respectively), did not show differential expression in response to ABA. These two genes are part of Subfamily I (Fig 3) which contain the ABA-independent SnRK2 proteins. Subfamilies II and III contain the ABA-dependent SnRK2 proteins [57]. Conversely, *IbSnRK2*.*5* is significantly down-regulated during SR formation (Figs 5 and 6) and is a member of the ABA-dependent Subfamily II. *IbSnRK2*.*5* may therefore be an important negative regulator of sweet potato SR formation in response to ABA.

Except for *IbPYL2*, all the differentially expressed *IbPYLs* were down-regulated in response to ABA, MeJA, and/or SA (Fig 7). A similar expression pattern was observed in potato, where several *StPYLs* had lower expression after ABA treatment [58]. Interestingly, most of these *StPYLs* showed up-regulated expression in response to auxin [58].

The high degree of connectivity in the predicted PPI network suggests that these proteins regulate multiple developmental pathways in a highly coordinated manner. Several *IbPYL*, *IbPP2C*, and *IbSnRK2* genes were differentially expressed in transgenic sweet potato plants that had stronger low-temperature stress tolerance than wild-type plants [59]. Solis et al. [60] found an *IbAREB* gene was up-regulated during drought stress. Additionally, Wang et al. [61] found that *IbABF4* confers resistance to several abiotic stresses in sweet potato and this gene was used to investigate drought tolerance in several Caribbean varieties of sweet potato [62]. *IbPYL8* forms part of a complex that mediates ABA-dependent drought responses [63]. The presence of CREs involved in stress and defence responses in the promoter sequences of the *I*. *batatas* ABA signalling genes (S2 Table) suggests that the proteins that are encoded by these genes are vital to the plant’s responses to various biotic and abiotic stresses.

This study lays a comprehensive framework that can be used for future studies on the functional validation of these genes. The well-studied *AtSnRK2*.*-2*, *-3*, and *-6* are considered core components of the *A*. *thaliana* ABA signalling network [64] so future experiments are required to determine if this is also true of the *I*. *batatas* homologs, *IbSnRK2*.*3* and *IbSnRK2*.*6*. The predicted STRING PPI network indicated that IbSnRK2.3 and IbSnRK2.6 are the only members of this gene family that have high confidence interactions (confidence score > 0.7) with both PYL and ABF proteins (Fig 9), as opposed to the other SnRK2 proteins which either showed medium or low confidence interactions with PYL proteins. Additionally, the Subfamily II AtSnRK2 proteins (AtSnRK2.7 and AtSnRK2.8) regulate drought-responsive genes, such as AtABFs, to enable drought tolerance [65]. The high sequence conservation in the SnRK2 family suggests that there may be some conservation of function as well. AtAREB1, AtAREB2, and AtABF3 are Subfamily I members that are thought to be master regulators for ABA-dependent expression under water stress [66]. The corresponding *I*. *batatas* homologs (IbABF-1, -2, -3, and -4) are likely to also be involved in similar functions, as already experimentally shown for IbABF4 [61]. Several *MeABF*, *MePYL*, and *MeSnRK2* genes were up-regulated in cassava during multiple abiotic stresses and the sweet potato homologs likely have similar roles [67, 68].

The correlations between the *in silico* and qRT-PCR datasets give further support for the importance of these genes during storage organ formation. A limitation of using datasets from different cultivars is that there are more factors involved in the variations in gene expression that were observed so that a specific reason for the differences in gene expression could not be identified.

## Conclusions

In this study, 9 *IbABF/ AREB/ ABI5*, 11 *IbPYL*, and 8 *IbSnRK2* genes were identified and characterized from the *I*. *batatas* genome. The physicochemical properties, domain organizations, and phylogenetic analyses of these sequences revealed that these sequences are highly conserved. There were numerous *cis*-regulatory elements involved in light responsiveness, hormone responsiveness, developmental responses, and defence and stress responses in the promoter sequences of these genes. *In silico* expression analyses revealed that several of these genes were differentially expressed during SR initiation and development. Similar expression patterns were observed with qRT-PCR. The predicted PPI network for these proteins indicated that these ABA signalling proteins form a highly connected network. The up-regulation of *IbABF3*, *IbABF4*, *IbDPBF3*, *IbDPBF4*, *IbPYL4*, *IbSnRK2*.*1*, and *IbSnRK2*.*2* during SR development makes them ideal candidates for further investigations on their roles during SR initiation.

## Supporting information

S1 Table List of primers used in qRT-PCR reactions. (DOCX)

S2 Table CREs present in the promoter sequences of ABA signalling genes. CREs in bold type are the broad category that encompasses several CREs with the same function. The boxes are coloured in different shades of red, with increasing intensity representing increasing numbers of CRES.
(XLSX)

S3 Table qRT-PCR Ct values and expression calculations. (XLSX)

S1 File Complete CDS and protein sequence for g9406 and g25480 used in this study. (DOCX)

S1 Fig Gene Ontology (GO) analysis of *I*. *batatas* ABF/AREB/ABI5, PYL, and SnRK2 proteins. The top 80 enriched GO terms are shown.
(TIF)
