# Supplementary material for: Characterization and expression analysis of SnRK2, PYL, and ABF/ AREB/ ABI5 gene families in sweet potato
Source: PLoS One. 2023 Nov 3;18(11):e0288481. doi: 10.1371/journal.pone.0288481 (PMC10624305; doi:10.1371/journal.pone.0288481)
Supplement: S1 Table — (DOCX) [file pone.0288481.s001.docx]

| **Name** | **Forward Primer (5’**–**3’)** | **Reverse Primer (5’**–**3’)** |
| --- | --- | --- |
| **IbABF2** | GGGATGGGTATGGTAGGTTTG | CGGAAGATGTATCACCGTTACT |
| **IbABF4** | ACCACAGCCAGAAGGTAATG | CCTTCCCAAGTCCGCTAAAT |
| **IbDPBF1/ABI5** | GATTTACTCGCTCACCTTGGA | GCGGTCCAAATGCTGTTAAG |
| **IbPYL4** | CAGATTCCGAACACCACAAGA | CACGATGGTGTCAACGAAGA |
| **IbSnRK2.2** | GGAAGTAGTAGGTGGTGATGATG | TGGGTTGGGTGATGTGATATT |
| **IbSnRK2.5** | GCTGTTTCCTATTATGCAATTCTGGG | ACTTTTCCTCCATCGCAACTCA |

**S1 Table. List of primers used in qRT-PCR reactions.**
