# Supplementary material for: Characterization and expression analysis of SnRK2, PYL, and ABF/ AREB/ ABI5 gene families in sweet potato
Source: PLoS One. 2023 Nov 3;18(11):e0288481. doi: 10.1371/journal.pone.0288481 (PMC10624305; doi:10.1371/journal.pone.0288481)
Supplement: S1 File — (DOCX) [file pone.0288481.s004.docx]

**S1 File. Complete CDS and protein sequence for g9406 and g25480 used in this study.**

>g9406

MVLEEKMEAEYIERFHKHQPTEYQCSSIVTNHIKAPTDIVWSLVRRFDQP

QKYKPFVSRCTVLGDLAIGSVREVDVKSGLPATTSTERLELLDDEEHILG

IRIVGGDHRLKNYSSVITVHPEIIDGRPGTLVIESFLVDVPEGNTRDDTC

YFVNALINCNLKALADVSERMAIQDGNGIASTTVSWASSAYIKP

>IACB01087057.1 TSA: Ipomoea batatas mRNA, contig: comp146111_c0_seq1, strain: Kokei 14, transcribed RNA sequence

CTATTTTATTATTAATTAAAATGGTTAGTGTTACTTGTCGGTAGACCCTGACAAGGACAAAATAGGGAGT

ATAATAATATAATATACTCCGTAATATGCTTTGCAGTCTGCAATAAAGATCTTTCACTTCTGGTTTTATA

TATCATAGGCGTAGGTGATAGAGTAGACGACTTTCTTTCACTTTAACCACGCAAAGATCGTATCTTTTTC

CTCGGTGAATTTTGGGTTTAAGCTAAGCTGTCTTCTTTTCTGTGCTTTTCCTCCGGGGAATTGATGGTTT

TGGAAGAGAAAATGGAAGCAGAGTACATAGAGAGATTCCACAAGCATCAGCCTACAGAGTATCAGTGTTC

TTCCATTGTTACGAACCACATCAAAGCTCCCACTGATATTGTTTGGTCACTGGTGAGGAGGTTTGATCAG

CCACAGAAGTATAAGCCATTTGTTAGCAGGTGTACAGTTTTGGGTGATCTTGCAATTGGGAGTGTTAGAG

AGGTAGATGTGAAGTCAGGACTTCCTGCTACAACCAGCACTGAAAGGTTGGAACTTCTTGATGATGAGGA

GCATATCCTTGGGATCAGGATTGTTGGTGGTGATCACAGACTGAAGAACTACTCTTCAGTCATTACAGTC

CATCCAGAGATAATTGATGGGAGACCAGGAACACTGGTGATCGAGTCATTTTTGGTAGATGTGCCTGAAG

GGAACACTCGAGACGATACCTGCTACTTTGTGAATGCGCTTATCAACTGTAACCTGAAAGCTTTGGCAGA

TGTCTCGGAGAGAATGGCCATTCAGGATGGGAATGGGATTGCATCTACGACTGTGAGCTGGGCATCTAGT

GCCTATATTAAGCCGTAAAGGCTTTTGTTTTGAGATTGGGAAGACTGCTTGAAGCAGATCCACTTTTCCA

TTTCAATCTAATTATATTTATAGTTCCGGTCCTCTAAAACCGCTGGAGTTTCCACTGTGCTGTAGGTTAA

GCTTTTGATTATTAGTATCTGTAAATATAATATATGCATAGGAACATGTTGGTCCTGTGGAGACACTATT

ATGCCAATCTAGTGTAGACCACTTTCCTTAGTTTGAGTAAATAATCAGAGTGATTTGACCTTCCTTTGTG

GTAGGTTTGATAGTTGTATCATTTTTTCATGCTGCTTTAATTTAGCTTTTGATTATTGTTCATGCTCAAG

AACAGTACACTGCACTTAAAATGGGGGACAGACAATGTGAATATCTTGAAGCAGTAGAAGCAAAAAAAAA

ATTAACCTGCCTGAGTCTCTGTGTGTACTCGCTCTTTAGTTACATATACATCAAAGCTTCTGGATTTCCA

TCAATTTTCACTATCATATTTACAGCTTGAATTTGTAACTTGAATGCTACAGGCCGAATAGTTCTGCTAC

TCTCGAACATCTATGGTGATGCTTAAATCCCTACCAGAAGTCATTGCAGGAGCACTTACCATCCTTAAAA

TGATGAAAACGCGCGCCATCTTTCAGGAAAATCTCCCTCTTCCTCAACAGGGACATATATTTTGCCACCG

TGTGACAGTCGGAGCAAACTTTTGTGTTCTTAATCACCCGAACTGGGCTGCAACTTCTGCTTTTGATTAG

ACCGTATGTAATTGCCAGTTTCTCTGTGTGATGAAGCAACTCCTTTTCTGGTTCATCAATGTTCTGATAA

ACGCATTTAGTATCAGCCACATACCCCAACAGCTTCATCTCGGAAATCAATTTGTACAGCTCAAAATAGA

TGATTCCTTCATCTGGATGTGGTTTTCCAACTGCAGAGAAGATGTGAATCTTCTGATCAATTTGTAGCCA

GCTCCAAACATTTTCTGTTCTCGCTCCCACTGTATCCATCATCTCTTTTATGCGGCTCACATCCTGCCC

> g25480

MPSALQLHQRGSAAAAAGVGSVYKQAVQAAARWMIPVSISVPEHVLQYHT

HAVGAGQCCAAVVQEVAAPLEAVWRLVRRFDKPQAYKHFLKSCHVIVGDG

DVGTLREVRVVSGLPAASSTERLEILDDEKHVLSFAVVGGDHRLNNYRSV

TTLHPHHRNNTTVVVESYVVDVPPGNTNDETCVFVDTIVRCNLQSLAQIA

ENSNNSPNQELKSHTTNTVIDVR

>GBZH01012134.1 TSA: Ipomoea batatas contig_34219 transcribed RNA sequence

CGGAGGGAGTATATTGTTAATTAACTCAGGAGATTGAAGAAAATCCCCCAAGCAAAAATAAAAGATTGAA

CAAAAAAAATACAAGAAATAATGTAGTATATATATATATGTGTTTGGTTACAAAACATGTGCATTCGAGA

TCTTCATCACACCCTCCAAATCTAATAACCAACCTTAGCTTTATAATCTTCAACGAACATCTATCACTGT

ATTGGTAGTATGACTTTTCAGTTCTTGATTTGGAGAGTTGTTGGAGTTTTCCGCGATTTGCGCTAGGGAT

TGAAGATTGCAACGTACGATGGTGTCGACGAAGACGCAGGTTTCGTCGTTGGTGTTGCCGGGGGGGACGT

CGACGACGTAGGACTCGACGACGACGGTGGTGTTGTTGCGGTGGTGGGGGTGGAGGGTGGTGACGGAGCG

GTAGTTGTTGAGGCGGTGATCGCCGCCGACGACGGCGAAGCTGAGCACGTGCTTCTCGTCGTCGAGGATC

TCGAGGCGCTCGGTGCTGGAGGCGGCGGGGAGGCCGGACACCACGCGCACCTCGCGGAGCGTGCCCACGT

CCCCGTCGCCGACAATGACGTGGCAGCTCTTCAGGAAGTGCTTGTACGCCTGCGGCTTGTCGAAGCGGCG

CACCAGCCGCCACACCGCCTCCAGCGGCGCCGCCACCTCCTGCACCACCGCCGCGCAGCACTGCCCCGCC

CCCACCGCGTGCGTGTGGTACTGCAGCACGTGCTCCGGCACCGATATCGACACCGGAATCATCCACCGAG

CCGCCGCCTGAACCGCCTGCTTATAAACACTCCCCACTCCAGCCGCAGCCGCCGCCGAGCCGCGCTGATG

AAGCTGAAGCGCAGAAGGCATCTCCCTATTCTTTTCTTCCTGCCTAGTTTTTCTTTATGGTTGAAAGGAC

ACTTATGAGAATGTCTTTATATATACACACACTATCATATGTTACGAATATAATATCATATAATTACGAT

CTTCGTCGACACCATCATCAATCATATAATGAATTATATATATAGTAATATAGTATAAGGATATGAATGG

GTTGATCTTTCTTTCATAGGTAGTAGAAGAAGAAACTAAA
