# Supplementary figures and images for: Characterization and expression analysis of SnRK2, PYL, and ABF/ AREB/ ABI5 gene families in sweet potato
Source: PLoS One. 2023 Nov 3;18(11):e0288481. doi: 10.1371/journal.pone.0288481 (PMC10624305; doi:10.1371/journal.pone.0288481)

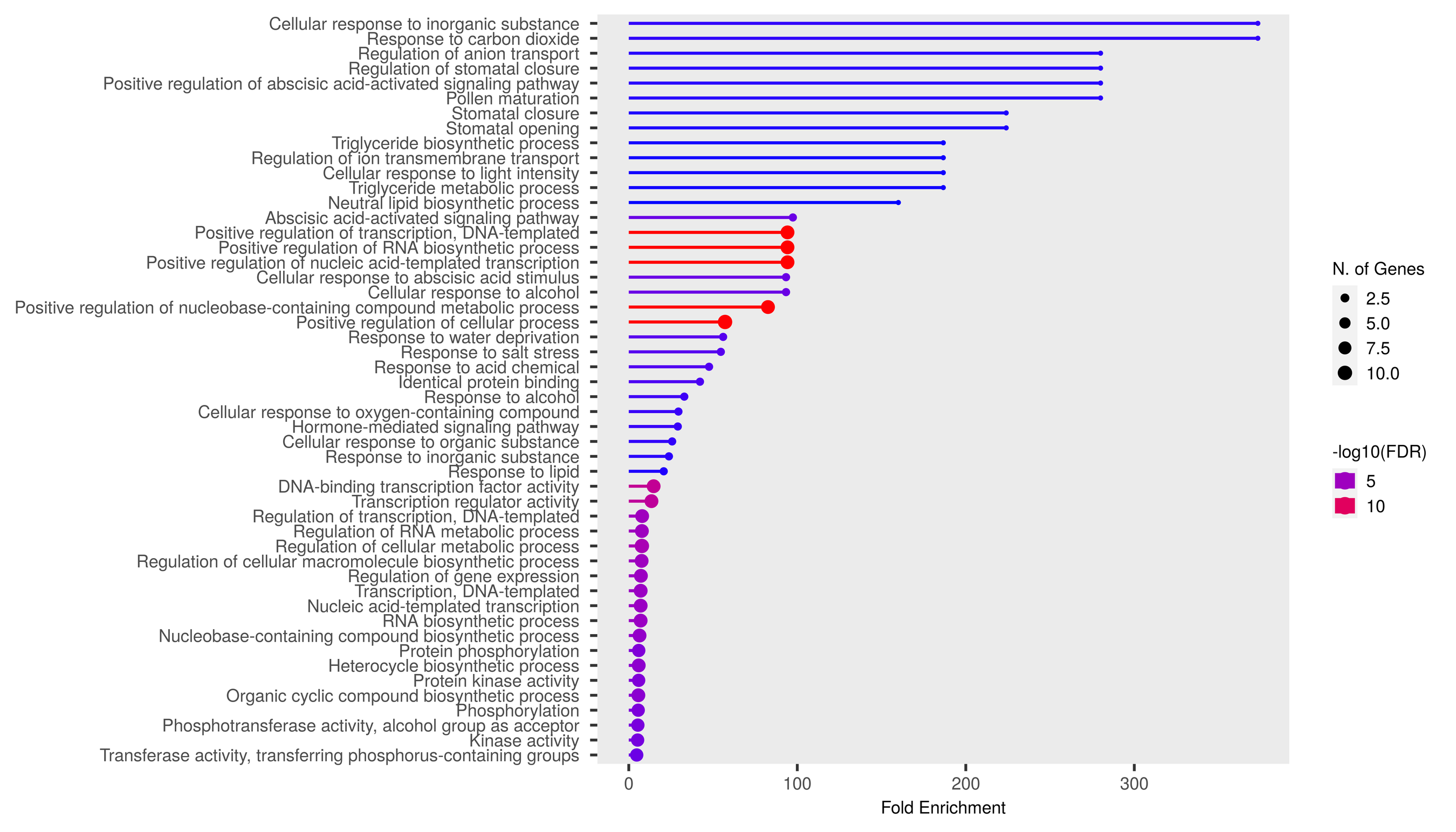

Supplement: S1 Fig — The top 80 enriched GO terms are shown. (TIF) [file pone.0288481.s005.tif]
